# Medical nutrition therapy in impaired glucose tolerance: A non-pharmacological strategy

**DOI:** 10.6026/973206300223192

**Published:** 2026-05-31

**Authors:** Sachin Parmar, Sethia Sethia, Priyaranjan Singh, Soumitra Sethia, Admira Fernandes, Shreyash Dwivedi, Durga Ahuja, Souryakant Varandani, Sapna Jain

**Affiliations:** 1 Department of Community Medicine, V.K.S. Government Medical College, Neemuch, Madhya Pradesh, India; 2 Department of Pediatrics, Sunderlal Patwa Government Medical College, Mandsaur, Madhya Pradesh, India; 3 Department of Community Medicine, Nand Kumar Singh Chauhan Medical College Khandwa, Madhya Pradesh, India; 4 Department of Community Medicine, Sunderlal Patwa Government Medical College, Mandsaur, Madhya Pradesh, India; 5 Department of Pharmacology, Nand Kumar Singh Chauhan Medical College Khandwa, Madhya Pradesh, India;; 6 Private Consultant, Vijay Naturopathy Center, Neemuch, Madhya Pradesh, India

**Keywords:** Impaired glucose tolerance (IGT), medical nutrition therapy (MNT), prediabetes

## Abstract

Impaired Glucose Tolerance (IGT) carries a significant risk of progression to Type 2 Diabetes Mellitus, yet structured nutritional interventions remain underutilized in Indian clinical settings. This is an interventional study at NSC GMC Khandwa on the efficacy of Medical Nutrition Therapy (MNT) in regulating the blood sugar level of 100 adult patients with Impaired Glucose Tolerance (IGT). The subjects were treated to a three month custom made MNT program which included systematic dietary consultation as well as individualized calorie schemes with regards to the level of occupational activity. The results of post intervention tests showed that dietary compliance (8 to 46), regular exercise (16 to 34) and caloric awareness (44 to 71) have improved significantly. As a result, the percentage of patients who were rated as being high-risk according to the random blood glucose levels reduced drastically, by 54 to 32. Thus, we show that structured MNT is effective to alter the lifestyle behaviour and is effective in the reduction of glycemic risk among prediabetic population.

## Background:

A 2-hour plasma glucose level of 7.8 -11.0 mmol/L after a 75g oral glucose tolerance test indicates hepatic and peripheral insulin resistance with progressive beta-cell dysfunction in impaired glucose tolerance (IGT), a prediabetic condition [1]. International Diabetes Federation estimates that 541 million IGT adults worldwide are undiagnosed, putting a strain on global health systems [2]. IGT patients have a 51% annual risk of developing open type 2 diabetes mellitus (T2DM) without treatment, so early treatment is crucial [3]. A registered dietitian nutritionist provides medical nutrition therapy (MNT), an evidence-based nutrition care process that involves dietary assessment, intervention and follow-up to meet glycaemic, weight and cardiovascular goals. There is strong evidence that MNT lowers HbA1c by up to 2.0% in T2DM and that it improves glucose tolerance in prediabetics similar to pharmacological agents in early dysglycemia. Before pharmacotherapy, all adults with prediabetes should receive individualised MNT [4]. An intensive lifestyle program (150 minutes/week of physical activity) focused on 7% weight loss was effective in reducing T2DM risk by 58% in adults with IGT over three years [5]. The Finnish Diabetes Prevention Study (FDPS) reported a 58-percentage decline in diabetes incidences due to individualised dietary counselling on fat and fibre intake, supporting the primary role of nutritional modification in IGT management [6]. The Da Qing IGT and Diabetes Study, the first large-scale RCT on IGT, found a 3347-percentage risk reduction with diet and physical activity over 6 years and long-term protection at 20 years [7].

The Indian Diabetes Prevention Programme (IDPP-1) found that lifestyle change prevented T2DM in 28.5% of IGT Asian Indians, highlighting the importance of MNT-based interventions in South Asia [8]. Different mechanisms control IGT depending on diet. Reducing refined carbohydrate intake and replacing high-GI foods with low-GI ones reduces postprandial glucose excursions and glucotoxic beta-cell damage in IGT patients [9]. Dietary fibre, especially whole grains, legumes and non-starchy vegetables, increases insulin sensitivity and slows IGT progression and is a key component of MNT treatment [10]. A meta-analysis of lifestyle interventions in adults with IGT found a standardised mean of treatment effect of -0.27 (95% CI: -0.38 to -0.15) in fasting plasma glucose and -0.53 in 2-hour plasma glucose after structured dietary intervention [11]. There is strong international evidence that MNT can be translated into routine clinical care for IGT patients, but dietitians, dietary counselling infrastructure, patient compliance and standardised context-specific IGT nutrition guidelines are still low in India. To fill this evidence gap, India-specific and context-sensitive MNT trials targeting IGT populations are urgently needed [8]. Medical Nutrition Therapy (MNT) is one of the key evidence-based programs, which modifies the underlying metabolic dysfunction in Impaired Glucose Tolerance (IGT) a prediabetic state, by prescribing a specific dietary change to reduce the development of Type 2 Diabetes Mellitus (T2DM). Therefore, it is of interest to study role of medical nutrition therapy in better Maintenance of blood sugar level in impaired glucose tolerance patients.

## Materials and Methods:

## Design and setting of the study:

This was an interventional educational study that was carried out in duration of three months at NSC Government Medical College, Khandwa, and Madhya Pradesh. The aim of the research was to compare the effectiveness of Medical Nutrition Therapy (MNT) in the improved management of the blood sugar levels in patients with Impaired Glucose Tolerance (IGT).

## Population and sample size of the study:

One hundred IGT patients were recruited in the study according to the set inclusion and exclusion criteria. The population used in the study was patients aged between 30-50 years with 62 men and 38 women.

## Inclusion criteria:

[1] Included patients with Impaired Glucose Tolerance (IGT) between 30 and 50 years old

[2] Participants who are willing to give a verbal informed consent.

## Exclusion criteria:

[1] Non-consenting participants

[2] Participants who do not have complete or missing information

[3] Patients with diabetic values of HbA1c during the enrolment.

## Data collection:

Each participant was interviewed with the help of a pre-tested, semi-structured questionnaire that contained all the questions to evaluate the existing lifestyle practices, dietary habits, physical activity, smoking behaviour, alcohol intake and consciousness of caloric value and intake after providing a verbal informed consent. This was the pre-intervention assessment.

## Intervention:

According to the results of pre-intervention assessment, all patients enrolled in the program were assigned to the tailored Medical Nutrition Therapy that included the structured dietary counselling and a personalised calorie plan depending on their daily level of occupational activity;

[1] Hard workers: 3200-3900 kcal/day

[2] Moderate workers: 2600-3200 kcal/day.

[3] Office employees: 2200 to 2600 kcal/day.

The participants were informed about the macronutrient ratio, low GI diets, food portions, the timing of meals and the relevance of exercises in managing the glycaemic index. Dietary counseling was pegged on WHO and National dietary guideline on the management of IGT.

## Post-intervention assessment:

The same semi-structured questionnaire that had been pretested was again given to all the participants at the end of three months to assess the changes in diet, physical exercise, behavioural changes and blood glucose levels after MNT.

## Outcome variables:

The major findings of the study were:

[1] Lifestyle and dietary change (diet compliance, exercise, smoking, alcohol consumption and caloric awareness) pre- and post- intervention.

[2] Change in risk stratification, pre-intervention and post-intervention based on random blood glucose levels (high risk: 121-125 mg/dl; moderate to low risk: 120 or less)

## Statistical analysis:

Microsoft Excel and SPSS version 22.0 were used to enter and analyse the data. Frequencies and percentages were used to present categorical variables. The chi-square test was used in evaluating the relationship between pre- and post-intervention categorical variables. The p-value of below 0.05 was taken to be statistically significant.

## Ethical considerations:

This research was done upon the consent of the Institutional Ethics Committee (IEC). All participants gave informed consent verbally before enrolment. Confidentiality and anonymity of the patients were observed during the study.

## Results and Discussion:

Table 1 is the pre- and post-intervention comparison of behavioural habits in 100 IGT patients. Before the intervention (pre-MTN), 8% of the patients observed an adherence to a diet regimen; it significantly increased to 46% after the intervention, which is an improvement of 82.6%. The current research followed dietary prescription based on occupational activity, with hard workers (3200-3900 kcal/day), moderate workers (2600-3200 kcal/day) and sedentary workers (2200-2600 kcal/day) as the occupational activity. This is an important increase in dietary adherence that is consistent with the consensus report of Evert *et al.* [1] that showed that individualized MNT provided by a registered dietitian lowers HbA1c by 0.3-2.0% in prediabetes and greatly enhances dietary adherence when caloric goals are adjusted to patient-specific activity levels. Guess [9] further added that a cornerstone intervention in IGT is structured dietary advice, focusing on macronutrient composition and caloric adequacy with a high level of correlation of adherence with face-to-face counselling frequency. The proportion of regular exercise increased by 52.94 relative (52.94 x 100) as the proportion of regular exercise participation increased by 16% to 34% between pre-intervention and post-intervention (8.641; p = 0.0033) (Table 1). This statistically significant difference is congruent with the results of a meta-analysis by Zhang *et al.* [11] which found that physical interventions in IGT were independently related to a significant reduction in the 2-hour plasma glucose levels (SMD: -0.42; 95% CI: -0.63 to -0.20), which once again supports the idea that the adherence to exercise is the key determinant A network meta-analysis of Zhang *et al.* [12] reported moderate-intensity aerobic exercise combined with resistance training to have the most favorable results in reducing fasting blood glucose, HbA1c and BMI in prediabetes and that any form of exercise yielded better results in terms of glycaemic control compared to no exercise, which confirms the positive trend in the current study. There was no alteration in the smoking behaviour of the patients after the interventions (p = NS) and there was a nonsignificant but marginal decrease in alcohol consumption, 11.11% (p = 0.706) (Table 1). These results underscore the fact that altering addictive behaviours with the help of dietary counselling alone is not an easy task as previously reported. In the Indian Diabetes Prevention Programme (IDPP-1), Ramachandran *et al.* [8] also reported that lifestyle modification programmes were inadequate to achieve smoking cessation among Asian Indian IGT patients and that supported behavioural counselling module, with nutritional intervention should be incorporated to contain tobacco and alcohol use as independent cardiometabolic risk factors in the management of IGT. Knowledge of caloric value and intake increased significantly between 44 percent before intervention and 71 percent after intervention by 38.02 which was very significant (p = 0.0001) (Table 1). This observation is corroborated by the evidence presented by Evert *et al.* [4] who had found that when nutrition education based on caloric awareness is delivered in the structured form, self-management behaviours and dietary quality scores are positively influenced in adults with prediabetes. A groundbreaking RCT by the DPP Research Group led by Knowler et al. [5] showed that intensive dietary education and self-monitoring of caloric intake were the keys to the 7% weight loss goal that lowered the incidence of T2DM by 58% among IGT people, which once again testifies to the critical role of caloric literacy as a modifiable predictor of glycaemic risk. Table 2 shows the stratification of the patients in terms of the random blood glucose pre- and post- intervention of the MNT. The proportion of patients who were in the high-risk category (random blood glucose 121-125 mg/dL) was 54% and in the moderate-to-low risk category (≤120 mg/dL) only 46%. The proportion of high-risk patients reduced to 32% and moderate-to-low risk group grew to 68% after the intervention, 22% of the participants of the study became moderate to low-risk (p = 0.0017). This statistically significant negative change in glycaemic risk category after MNT is in agreement with the results by Jiang *et al.* [13] whose meta-analysis of 13 RCTs (13,376 participants, 6,058 in their sample) of 13 RCTs of 13 studies indicated that lifestyle interventions significantly decreased 2-hour. A large RCT, the Finnish Diabetes Prevention Study by Tuomilehto *et al.* [6] which involved the use of dietary counselling based on individualised dietary therapy in IGT patients, revealed that glycaemic benefits were the most noticeable when the dietary counselling was focused on the reduction of fat intake and an increase of fibre intake, with the most significant improvement in glycaemic benefits observed in patients who achieved. In a short-term structured dietary and exercise intervention on 542 prediabetic patients, Wang *et al.* [14] found that even short-term structured dietary interventions over six months induced significant improvements in values of FBG and 2-hour OGTT and seven-and-one-half-year follow-up established that sustained delay of diabetes progression was indeed present a fact which goes a long way to give credence to the clinical. The size of the glycaemic risk prevention in the present case is corroborated with the Da Qing IGT and Diabetes Study in which diet-only intervention led to a decrease in the incidence of diabetes by 33 per cent over six years in IGT patients, demonstrating that dietary modification alone is independently effective in generating significant, quantifiable glycaemic risk reduction even in the absence of pharmacotherapy. Combined, the current results support the evidence base that the structured MNT, using a better dietary compliance, caloric awareness and physical activity is a statistically significant and clinically useful strategy to reduce glycaemic risk in IGT patients at the community and district hospital level. Barrea *et al.* [15] Low-energy and very-low-energy diets, characterized by moderate to severe caloric restriction, respectively, have also been associated with improvements in cardiometabolic markers and glycaemic regulation.

## Conclusion:

Individualised Medical Nutrition Therapy, will benefit people with Impaired Glucose Tolerance by improving dietary adherence, exercise habits and caloric awareness. Such behavioural improvements were associated with clinically relevant reductions in random blood glucose levels, moving many patients into lower risk categories. Overall, this organised, non-medicated approach is highly efficient and effective in delaying or preventing the progression from prediabetes to type 2 diabetes.

## Figures and Tables

**Table 1:** Change in habit pre- and post-intervention

	**Diet Regimen Followed**		**Regular Exercise**		**Smoking Behaviour**		**Alcohol Intake**		**Awareness of calorie value & intake**	
Phase		No	Yes	No	Yes	No	Yes	No	Yes	No
Pre Intervention	8	92	16	84	12	88	18	82	44	56
Post Intervention	46	54	34	66	12	88	16	84	71	29
Improvement post intervention	82.60%		52.90%		No Change		11.11%		38.02%	
χ^2^test	36.631		8.641		0		0.142		14.916	
p Value	< 0.0001		0.0033		NS		0.706		0.0001	

**Table 2:** Risk assessment based on blood sugar level

**Phase**	**High Risk**	**Moderate To Low Risk**	**Total**
	(Random blood glucose level 121-125 mg/dL)	(Random blood glucose level ≤120 mg/dL)	
Pre-intervention	54	46	100
Post-intervention	32	68	100
